# Introduction of pyrrolidineoxy or piperidineamino group at the 4-position of quinazoline leading to novel quinazoline-based phosphoinositide 3-kinase delta (PI3Kδ) inhibitors

**DOI:** 10.1080/14756366.2018.1444608

**Published:** 2018-03-14

**Authors:** Minhang Xin, Weiming Duan, Yifan Feng, Yuan-Yuan Hei, Hao Zhang, Ying Shen, Hong-Yi Zhao, Shuai Mao, San-Qi Zhang

**Affiliations:** Department of Medicinal Chemistry, School of Pharmacy, Health Science Center, Xi’an Jiaotong University, Xi’an, P.R. China

**Keywords:** PI3Kδ inhibitors, 4-pyrrolidineoxy, 4-piperidineamino, selectivity, anti-proliferation

## Abstract

Phosphoinositide 3-kinase Delta (PI3Kδ) plays a key role in B-cell signal transduction and inhibition of PI3Kδ was confirmed to have clinical benefit in certain types of activation of B-cell malignancies. Herein, we reported a novel series of 4-pyrrolidineoxy or 4-piperidineamino substituted quinazolines, showing potent PI3Kδ inhibitory activities. Among these compounds, **12d**, **14b** and **14c** demonstrated higher potency against PI3Kδ with the half maximal inhibitory concentration (IC_50_) values of 4.5, 3.0, and 3.9 nM, respectively, which were comparable to idelalisib (IC_50_ = 2.7 nM). The further PI3K isoforms selectivity evaluation showed that compounds **12d**, **14b** and **14c** have excellent PI3Kδ selectivity over PI3Kα, PI3Kβ, and PI3Kγ. Moreover, compounds **12d**, **14b** and **14c** also displayed different anti-proliferative profiles against a panel of four human B cell lines including Ramos, Raji, RPMI-8226, and SU-DHL-6. The molecular docking simulation indicated several key hydrogen bonding interactions were formed. This study suggests the introduction of pyrrolidineoxy or piperidineamino groups into the 4-position of quinazoline leads to new potent and selective PI3Kδ inhibitors.

## Graphical abstract

**Figure:**
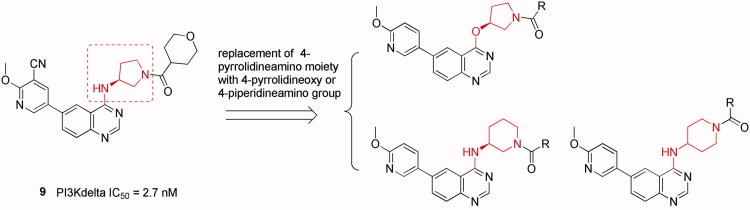

## 1. Introduction

**Figure 1.**
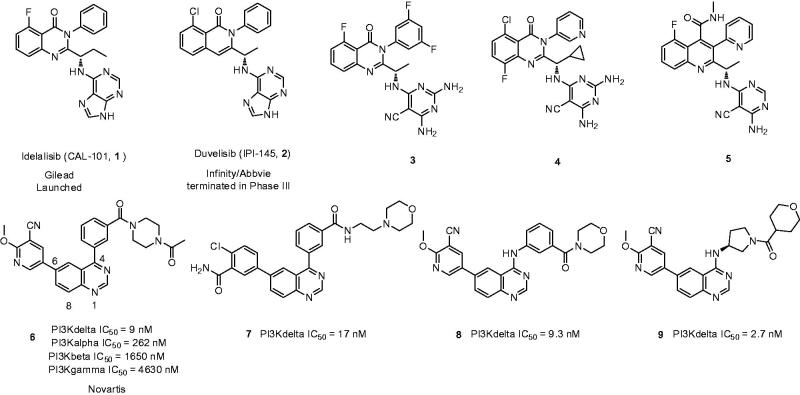
Representative structures for previously reported potent PI3Kδ inhibitors.

Phosphoinositide 3-kinases (PI3Ks) play a pivotal role in multiple cellular functions including cell growth, development, migration, angiogenesis, and survival^1^. Upon stimulation of cytokine signaling, PI3Ks function as an intracellular secondary messenger transforming phosphatidylinositol 4,5-bisphosphate (PIP2) into phosphatidylinositol 3,4,5-trisphosphate (PIP3) via phosphorylation catalysation, thereafter activation of the downstream signal transducer (Akt, mTOR) and subsequent activator of transcription^2^. There are four PI3K isoforms validated, including PI3Kα, PI3Kβ, PI3Kγ, and PI3Kδ. PI3Kα and PI3Kβ are ubiquitously expressed in multiple cells whereas PI3Kδ and PI3Kγ are identified as predominant expression in hematopoietic cells^3^. In particular, PI3Kδ is found responsible for the B-cell receptor (BCR) signaling downstream transduction and constitutive studies show activation of BCR signaling pathway is a hallmark of B-cell malignancies such as chronic lymphocytic leukemia (CLL), follicular lymphoma (FL), mantle cell lymphoma (MCL), small lymphocytic lymphoma (SLL), diffuse large B-cell lymphoma (DLBCL), and indolent non-Hodgkin’s lymphoma (iNHL)^4^. Therefore, PI3Kδ is thought as suitable target for the potential treatment of certain B-cell malignancies, as well as immunologic disorders (due to its specific role in controlling immune cell function)^5^. Notably, small molecules selective PI3Kδ inhibitor idelalisib (Compound **1**) has been recently approved by Food and Drug Administration (FDA) for treatment of CLL, FL, and SLL, which solidify the therapeutic concept of PI3Kδ inhibitor (Figure 1)^6^ ^,^ ^7^.

**Figure 2.**
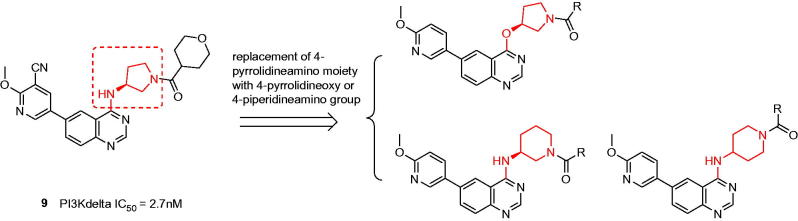
Design of the 4-pyrrolidineoxy and 4-piperidineamino substituted quinazolines as PI3Kδ inhibitors.

Despite the first-in-class approved, potent and oral selective PI3Kδ inhibitor, idelalisib was tagged with black-box warning and demonstrated struggling with severe adverse events in the later clinical validation^8^. Therefore, there is an urgent need to develop second generation PI3Kδ inhibitor with lower toxicity and fewer side effects. Duvelisib (Compound **2**), another potent PI3Kδ inhibitor, shared chemical similarity to idelalisib, however, this was recently terminated in the phase III clinical trials due to underneath efficiency. Many other analogues derived from the chemical structure of idelalisib were recently reported and showed strong PI3Kδ efficacy and selectivity, for instances Compounds **3** (PI3Kδ: half maximal inhibitory concentration (IC_50_) = 2.2 nM), **4** (PI3Kδ: IC_50_ = 1.0 nM), and **5** (PI3Kδ: IC_50_ = 4.6 nM)^9–11^. Nevertheless, our drug discovery efforts are engaged into the development of PI3Kδ inhibitors with novel and distinct chemotypes. Recently, we reported a new series of potent PI3Kδ inhibitors, chemically featured by a quinazoline scaffold and a 6-benzamide moiety such as Compound **7** (PI3Kδ: IC_50_ = 17 nM)^12^, derived from the Novartis’s patented Compound **6** (PI3Kδ: IC_50_ = 9 nM) with potent PI3Kδ inhibition and selectivity^13–15^. A subsequent structural modification was carried out and a series of 4-anilinequinazolines was synthesised, exemplified by Compound **8** (PI3Kδ: IC_50_ = 9.3 nM) showing improved PI3Kδ inhibition^16^. Later, further structural investigation by replacing the 4-aniline with a 4-pyrrolidineamino moiety led to a series of potent and selective PI3Kδ inhibitors, such as Compound **9** (PI3Kδ: IC_50_ = 2.7 nM), showing equivalence to idelalisib in our examination (Figure 1)^17^. Encouraged by these fantastic findings, we decided to develop a new series of quinazoline based PI3Kδ inhibitors by introducing functionalised pyrrolidineoxy or piperidineamino group at the 4-position of quinazoline instead of the pyrrolidineamino moiety. Herein, we disclose the synthesis, biological evaluation of this series of 4-pyrrolidineoxy and 4-piperidineamino substituted quinazolines as potent and selective PI3Kδ inhibitors (Figure 2).

## 2. Results and discussion

### 2.1. Chemistry

**Scheme 1.**
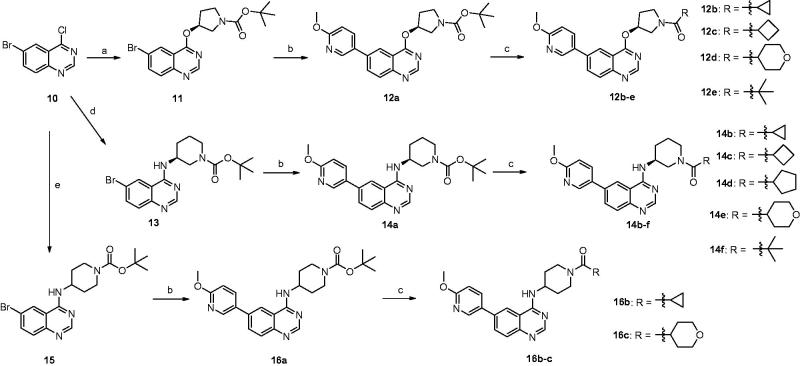
Reagents and conditions: (a) (S)-1-Boc-3-hydroxypyrrolidine, anhydrous THF, NaH, rt, overnight, 70%; (b) 6-methoxy-3-pyridinylboronic acid, Na_2_CO_3_, PdCl_2_ (dppf), DME/H_2_O, reflux, 4 h, 65–81%; (c) (i)TFA, CH_2_Cl_2_, rt, 2 h, 23–91%; (ii) diverse acids, DMF, HATU, DIPEA, rt, 12 h, 23–91%; (d) (S)-1-Boc-3-aminopiperidine, DMF, DIPEA, 90 °C, 6 h, 90%; (e) 1-Boc-4-aminopiperidine, DMF, DIPEA, 90 °C, 6 h, 52%.

The 4-pyrrolidineoxy and 4-piperidineamino substituted quinazoline derivatives were synthesised according to the synthetic routes outlined in Scheme 1. Treatment of 6-bromo-4-chloroquinazoline (Compound **10**) with (S)-1-Boc-3-hydroxypyrrolidine in the presence of sodium hydride (NaH) gave (S)-4-pyrrolidineoxyqui-nazoline (Compound **11)** in 70% yield, which was subsequently reacted with 6-methoxy-3-pyridinylboronic acid using Suzuki coupling condition to generate Compound **12a** ^18^ ^,^ ^19^. Compound **12a** reacted with TFA at room temperature to get rid of the tert-butyloxycarbonyl protecting group (Boc group) and then was acylated with diverse acids to afford Compounds **12**(**b–e**). Compounds **14**(**a–f**) and **16**(**a–c**) were prepared by employing the similar synthetic procedures^20^. Compound **10** was treated with (S)-1-Boc-3-aminopiperidine or 1-Boc-4-aminopiperidine to give intermediate Compounds **13** and **15**, respectively, which in turn underwent Suzuki coupling reaction, deprotection, and condensation to produce Compounds **14**(**a–f**) and **16**(**a–c**) successfully (Scheme 1).

### 2.2. PI3Kδ inhibitory activity for the title compounds

All the newly synthesised compounds were assessed for their PI3Kδ inhibitory activities and idelalisib was employed as the positive control. The 4-pyrrolidineoxy substituted quinazoline analogs were firstly examined and the results are shown in Table 1. It was found all the 4-pyrrolidineoxy substituted quinazoline analogues displayed significant PI3Kδ inhibitory activities under the concentration of 100 nM. The initial Compound **12a** bearing a (S)-4-(1-Boc-pyrrolidin-3-yl)oxy side chain showed an inhibitory ratio of 79% at the concentration of 100 nM, while replacement of the tert-butoxy group with cyclopropyl group (Compound **12b**: 90%) afforded enhanced PI3Kδ inhibitory activity, showing an IC_50_ value of 9.3 nM. Switch of the cyclopropyl group (Compound **12b**) to cyclobutyl (Compound **12c**: IC_50_ = 6.1 nM) and tetrahydro-2*H*-pyran-4-yl (Compound **12d**: IC_50_ = 4.9 nM) groups led to higher PI3Kδ inhibitory activity, whereas replacement with the branched tert-butyl (Compound **12e**: 53%) resulted in weak PI3Kδ potency. In the 4-pyrrolidineoxy subseries, Compound **12d** bearing tetrahydro-2*H*-pyran-4-yl side chain afforded the most potent PI3Kδ inhibitory activity, which was approximately equivalent to control drug idelalisib (IC_50_ = 2.7 nM; Table 1).

**Table 1.** PI3Kδ inhibitory activity of 4-pyrrolidineoxy substituted quinazolines^a^.


Compounds	R	PI3Kδ Inhibition (%)^b^	PI3Kδ IC_50_ (nM)^c^
**12a**		79	ND
**12b**		90	9.3
**12c**		96	6.1
**12d**		98	4.9
**12e**		53	ND
**1**	–	96	2.7

^a^All the data are shown as the mean for at least two experiments.

^b^PI3Kδ inhibition at the concentration of 100 nM.

^c^The IC_50_ values for PI3Kδ inhibition.

ND: not detected.

Subsequently, the 4-piperidineamino substituted quinazoline analogues were evaluated and the data are shown in Table 2. The initial (S)-4-(1-Boc-piperidin-3-yl)amino Compound **14a** showed weak PI3Kδ inhibitory activity with an inhibitory ratio of 51% at the concentration of 100 nM. However, replacement of tert-butoxy group with diverse cyclic aliphatic substituents afforded highly improved PI3Kδ inhibitory activity. Analogue of Compound **14b** bearing a cyclopropyl group gave an IC_50_ value of 3 nM, and analogue of Compound **14c** tailed with a cyclobutyl group showed almost comparable potency, with an IC_50_ value of 3.9 nM, whereas analogues of Compound **14d** with a cyclopentyl terminal and Compound **14e** containing a tetrahydro-2*H*-pyran-4-yl tail showed a little less potent PI3Kδ inhibition than that of Compound **14b**, with IC_50_ values of 8.7 and 5.2 nM, respectively. Again, an attempt to shift the cyclic group to non-cyclic alkyl group such as tert-butyl (Compound **14f**: 54%) resulted in PI3Kδ inhibition largely reduced. Otherwise, an exploration of changing the (S)-4-(piperidin-3-yl)amino side chain into 4-(piperidin-4-yl)amino group was also conducted, and three analogues were synthesised. However, unfortunately, Compound **16a** bearing a Boc group (Compound **16a**: 72%) and Compound **16b** with a cyclopropyl group (Compound **16 b**: 70%) showed moderate PI3Kδ inhibition, while Compound **16c** incorporated with tetrahydro-2*H*-pyran-4-yl group(Compound **16c**: 48%) produced unsatisfactorily weak potency. This suggested the spatial orientation of the tailed acyl substituents was critical for PI3Kδ inhibition, which was consistent to the structure-activity relationship of our previously reported 6-aryl substituted 4-anilinequinazoline series. In this preliminary PI3Kδ inhibition evaluation, three compounds **12d**, **14b**, and **14c** showed IC_50_ values beneath 5 nM, being approximately comparable to idelalisib, which were picked out for further evaluation.

**Table 2.** PI3Kδ inhibitory activity of 4-piperidineamino substituted quinazolines^a^.


Compounds	R	PI3Kδ Inhibition (%)^b^	PI3Kδ IC_50_ (nM)^c^
**14a**		51	ND
**14b**		94	3.0
**14c**		91	3.9
**14d**		91	8.7
**14e**		88	5.2
**14f**		54	ND
**16a**		72	ND
**16b**		70	ND
**16c**		48	ND
**1**	–	96	2.7

^a^All the data are shown as the mean for at least two experiments.

^b^PI3Kδ inhibition at the concentration of 100 nM.

^c^The IC_50_ values for PI3Kδ inhibition.

ND: not detected.

### 2.3. Isoform selectivity of the new PI3Kδ inhibitors

Based on the above preliminary PI3Kδ inhibitory activity results, Compounds **12d**, **14b**, and **14c** were subsequently evaluated for their selectivity among PI3Kα, PI3Kβ, and PI3Kγ. As shown in Table 3, all three compounds **12d**, **14b**, and **14c** showed much lower potency against other three PI3K isoforms than that of PI3Kδ, although they displayed moderate PI3Kα inhibition. Compound **12d** with an IC_50_ value of 4.5 nM against PI3Kδ demonstrated 11-fold, 131-fold, and 103-fold selectivity over PI3Kα, PI3Kβ and PI3Kγ, respectively, whereas Compounds **14 b** and **14c** displayed the similar PI3Kδ selectivity which were 12- and 15-fold over PI3Kα, 105- and 96-fold over PI3Kβ, and 34- and 31-fold over PI3Kγ, respectively. Moreover, it was noted that selectivity of compound **12d** against the PI3Kβ and PI3Kγ isoforms was much higher than idelalisib, although the poor selectivity against PI3Kα was observed (Table 3).

**Table 3.** Isoform selectivity of compounds against PI3K (p110α, p110β, p110γ, and p110δ)

	IC_50_ (nM)^a^			
Compounds	p110α	p110β	p110γ	p110δ
**12d**	50.4	592.5	467.6	4.9
**14b**	36.6	317.2	104.0	3.0
**14c**	58.9	375.9	121.1	3.9
**1**	306.4	120.1	139.4	2.7

^a^The IC_50_ values are shown as the mean for at least two experiments.

### 2.4. *In vitro* anti-proliferative assays of the new PI3Kδ inhibitors

Furthermore, Compounds **12d**, **14b**, and **14c** were tested for their anti-proliferative activities against four human B cell lines including Ramos, Raji, RPMI-8226, and SU-DHL-6with idelalisib and SAHA as reference compounds. As shown in Table 4, Compound **12d** exhibited most potent anti-proliferation against RPMI-8226 (IC_50_ = 44 nM) among these four cell lines, whereas Compound **14b** showed significantly potent anti-proliferative activity against Ramos, Raji, and SU-DHL-6, but moderate anti-proliferation against RPMI-8226 and Compound **14c** also showed strong anti-proliferativity against SU-DHL-6 with an IC_50_ value of 1.49 nM. It was found that the reference PI3Kδ inhibitor idelalisib displayed markedly anti-proliferative activity against SU-DHL-6, whereas another reference drug SAHA (vorinostat) afforded significantly anti-proliferation against Ramos, Raji, and RPMI-8226. In a word, three Compounds **12d**, **14b**, and **14c** as well as idelalisib were observed showing different anti-proliferative profiles in the four human B cell lines (Table 4).

**Table 4.** Anti-proliferative activities of new compounds *in vitro*

	IC_50_ (μM)^a^			
Compounds	Ramos^b^	Raji^b^	RPMI-8226^b^	SU-DHL-6^c^
**12d**	1.34	9.81	0.44	3.23
**14b**	1.34	0.81	8.66	1.04
**14c**	ND	ND	ND	1.49
**1**	>10	9.95	5.49	0.65
**SAHA**	0.52	0.97	0.66	ND

^a^The IC_50_ values are shown as the mean for at least two experiments.

^b^Anti-proliferative activities were determined by(3-(4,5-dimethylthiazol-2-yl)-2,5-diphenyltetrazolium bromide) tetrazolium (MTT) reduction method.

^c^Anti-proliferative activities were determined by CCK-8 method.

ND: not detected.

### 2.5. Molecular modeling study

**Figure 3.**
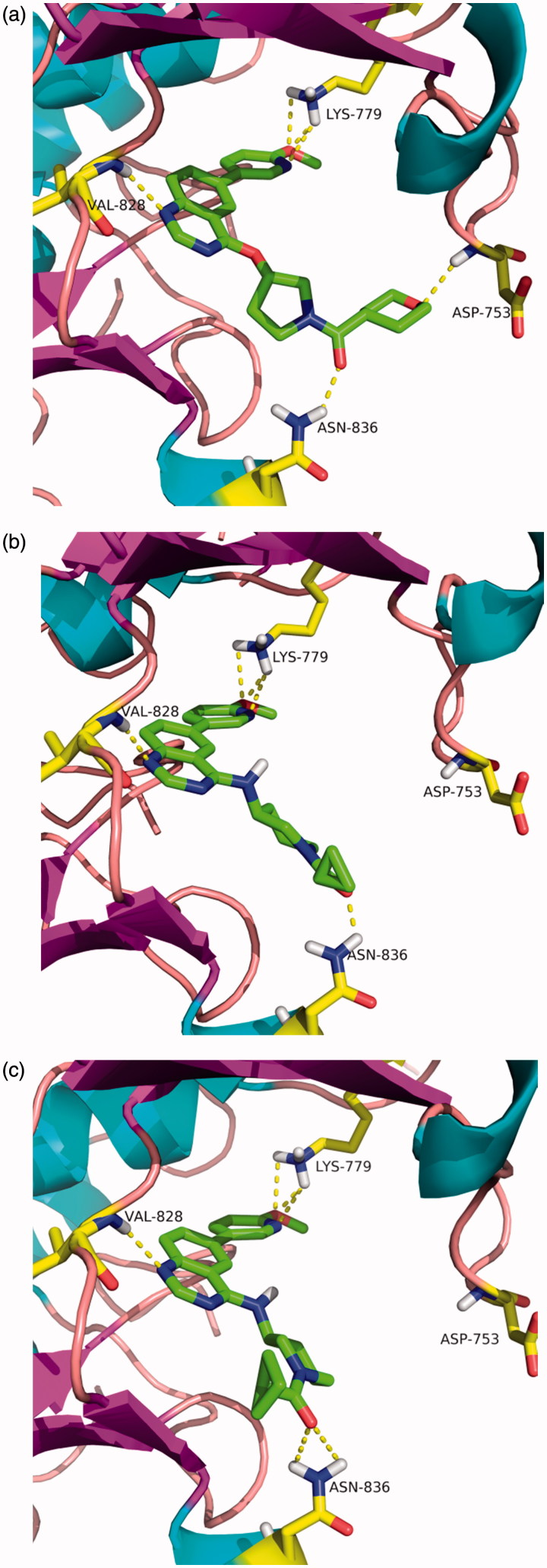
Molecular docking studies of Compounds **12d** (a), **14b** (b) as well as **14c** (c) into the site of PI3Kδ (PDB code: 2WXP). Compound is shown as sticks. Hydrogen bonds within 2.5 Å are shown as yellow dashed lines.

To further understand the potent PI3Kδ inhibition, molecular docking simulations of Compounds **12d**, **14b**, and **14c** within human PI3Kδ enzyme were performed. As shown in Figure 3, the docked pose of each Compound (**12d**, **14b** and **14c**) ma es the similarly favorable interactions with the PI3Kδ binding pocket of structure 2WXP as expected, namely, three key hydrogen bonds with the hinge residue, the quinazoline scaffold with Val828, the methoxypyridyl moiety with Lys779, as well as the carbonyl group with Asn836. Moreove r, it was observed that, although, the oxygen of the tetrahydro-2*H*-pyran-4-yl group in Compound **20a** formed an additional hydrogen bond with Asp753, it seemed to show little contribution for improving the inhibitory activity in this case (Figure 3).

## 3. Conclusion

In summary, we have synthesised and evaluated a novel series of quinazoline derivatives by introducing a functionalised 4-pyrrolidineoxy or 4-piperidineamino groups as potent PI3Kδ inhibitors. The structure-activity relationship (SAR) was discussed and many derivatives showed nanomolar PI3Kδ inhibitory activities, particularly, Compounds **12d**, **14b**, and **14c** demonstrating preferably potent PI3Kδ inhibitory activities with IC_50_ values of 4.5, 3, and 3.9 nM, respectively, approximately comparable to idelalisib (IC_50_ = 2.7 nM). Moreover, Compounds **12d**, **14b**, and **14c** showed excellent PI3Kδ isoform selectivity over PI3Kα, PI3Kβ, and PI3Kγ. These three compounds also displayed different anti-proliferative profiles against a panel of four human B cell lines. The molecular docking study indicated several key hydrogen bonding interactions formations, which may explain their higher PI3Kδ. This study suggests the introduction of pyrrolidineoxy or piperidineamino groups into the 4-position of quinazoline leads to new potent and selective PI3Kδ inhibitors
